# Molecular Mapping and Analysis of an Excellent Quantitative Trait Loci Conferring Adult-Plant Resistance to Stripe Rust in Chinese Wheat Landrace Gaoxianguangtoumai

**DOI:** 10.3389/fpls.2021.756557

**Published:** 2021-10-12

**Authors:** Yuqi Wang, Fengying Liang, Fangnian Guan, Fangjie Yao, Li Long, Xuyang Zhao, Luyao Duan, Yu Wu, Hao Li, Wei Li, Qiantao Jiang, Yuming Wei, Jian Ma, Pengfei Qi, Mei Deng, Youliang Zheng, Houyang Kang, Yunfeng Jiang, Guoyue Chen

**Affiliations:** ^1^Triticeae Research Institute, Sichuan Agricultural University, Chengdu, China; ^2^State Key Laboratory of Crop Gene Exploitation and Utilization in Southwest China, Sichuan Agricultural University, Chengdu, Sichuan, China; ^3^College of Agronomy, Sichuan Agricultural University, Chengdu, China

**Keywords:** adult-plant resistance, QTL, stripe rust, Chinese wheat landrace, genetic mapping, heterogeneous inbred family

## Abstract

The Chinese wheat landrace “Gaoxianguangtoumai” (GX) has exhibited a high level of adult-plant resistance (APR) to stripe rust in the field for more than a decade. To reveal the genetic background for APR to stripe rust in GX, a set of 249 F_6:8_ (F_6_, F_7_, and F_8_) recombinant inbred lines (RILs) was developed from a cross between GX and the susceptible cultivar “Taichung 29.” The parents and RILs were evaluated for disease severity at the adult-plant stage in the field by artificial inoculation with the currently predominant Chinese *Puccinia striiformis* f. sp. *tritici* races during three cropping seasons and genotyped using the Wheat 55K single-nucleotide polymorphism (SNP) array to construct a genetic map with 1,871 SNP markers finally. Two stable APR quantitative trait loci (QTL), *QYr.GX-2AS* and *QYr.GX-7DS* in GX, were detected on chromosomes 2AS and 7DS, which explained 15.5–27.0% and 11.5–13.5% of the total phenotypic variation, respectively. Compared with published *Yr* genes and QTL, *QYr.GX-7DS* and *Yr18* may be the same, whereas *QYr.GX-2AS* is likely to be novel. Haplotype analysis revealed that *QYr.GX-2AS* is likely to be rare which presents in 5.3% of the 325 surveyed Chinese wheat landraces. By analyzing a heterogeneous inbred family (HIF) population from a residual heterozygous plant in an F_8_ generation of RIL, *QYr.GX-2AS* was further flanked by *KP2A_36.85* and *KP2A_38.22* with a physical distance of about 1.37Mb and co-segregated with the *KP2A_37.09*. Furthermore, three tightly linked Kompetitive allele-specific PCR (KASP) markers were highly polymorphic among 109 Chinese wheat cultivars. The results of this study can be used in wheat breeding for improving resistance to stripe rust.

## Introduction

Stripe rust (yellow rust), caused by *Puccinia striiformis* f. sp. *tritici* (*Pst*), is among the most harmful and widespread obligate pathogens of common wheat (*Triticum aestivum* L.) worldwide (Knott, 1989; Wellings, 2011). In China, stripe rust prevailed for several times in large wheat-growing areas and this caused serious yield losses (Zeng and Luo, 2006; Chen et al., 2014; Han and Kang, 2018). Since the 1950s, four severe epidemics of wheat stripe rust have occurred in China in 1950, 1964, 1990, and 2002, resulting in yield losses of 6.0, 3.2, 1.8, and 1.4 million tonnes, respectively (Li and Zeng, 2000; Wan et al., 2004). The main cause of the outbreaks is the emergence of new virulent races that overcome the widely deployed resistance genes (Chen and Kang, 2017). At present, new virulent *Pst* race CYR34 appears and overcomes the widely deployed *Yr* genes. In the meantime, the simplification of *Yr* genes in commercial wheat cultivars have not changed yet though a lot of cultivars are used in wheat production. The most efficient and economical method of controlling the disease is the use of genetic resistance (Liu et al., 2017; Wang et al., 2019). Continuous improvement in the resistance of wheat cultivars to cope with evolving races of *Pst* is a high priority to control stripe rust (Manickavelu et al., 2016).

To date, more than 300 genes or quantitative trait loci (QTL) for stripe rust resistance on the 21 wheat chromosomes have been reported (Rosewarne et al., 2013; McIntosh et al., 2019). In general, these resistance genes and QTL can be classified into two major classes: all-stage resistance (ASR) and adult-plant resistance (APR). ASR usually confers complete resistance during all growth stages and is simple to select during breeding. However, most ASR genes are race specific and encode nucleotide-binding and leucine-rich repeat (NLR) proteins, and therefore are effective against only a subset of *Pst* races. With regard to the dynamic rust pathogen populations of the virulent races, only a small number of the characterized ASR genes, such as *Yr5* (Marchal et al., 2018) and *Yr15* (Klymiuk et al., 2018), are still widely effective against currently dominant *Pst* race groups in China (Sharma-Poudyal et al., 2013; Wu et al., 2018).

In contrast, APR is effective starting at adult-plant growth stages and typically provides a degree of partial resistance. Although a few APR genes are race-specific (Milus et al., 2015), a greater proportion of APR genes including *Yr18* (Krattinger et al., 2009), *Yr29* (William et al., 2003), *Yr30* (Hayden et al., 2004), and *Yr46* (Moore et al., 2015) is non-race-specific and provides durable resistance to *Pst*. Of the three APR genes cloned to date, *Yr18* encodes a putative ATP-binding cassette transporter (Krattinger et al., 2009), *Yr36* encodes a kinase domain and a lipid-binding domain (Fu et al., 2009), and *Yr46* encodes a predicted hexose transporter (Moore et al., 2015). These genes represent different protein families compared with classical ASR genes (the NLR family) and provide unique mechanisms effective against a broader range of pathogens. As an example, *Yr18* has been globally used as a component of durable rust resistance in breeding programs and no evolution of increased virulence has been observed for almost 100years (Krattinger et al., 2009). To achieve a high degree of durable resistance, combining multiple APR genes into the same background has been considered as an important strategy for improvement of stripe rust resistance in wheat breeding.

Chinese wheat landraces are farmer-developed and maintained as traditional cultivars in China. These landraces harbor rich genetic diversity for stripe rust resistance. Numerous stripe rust genes or QTL have been identified, such as *Yr1* (Bansal et al., 2009), *Yr18* (Krattinger et al., 2009), *Yr81* (Gessese et al., 2019), *YrYL* (Wu et al., 2016a), *YrBai* (Ma et al., 2015), *Yrqbc* (Cao et al., 2020), *QYr.caas-5AL* (Lan et al., 2010), *QYr.cau-6DL* (Zhang et al., 2017), *QYr.cau-2AL* (Wang et al., 2019a), *QYr.GTM-5DL* (Wu et al., 2020), and *QYr.AYH-5BL* (Long et al., 2021). Recently, our research program evaluated more than 1,000 Chinese wheat landrace accessions collected from all 10 agro-ecological zones (Zhou et al., 2017) for responses to stripe rust in the greenhouse and the field under inoculation with selected Chinese predominant races of *Pst* (Cheng et al., 2019; Long et al., 2019; Yao et al., 2019, 2020; Ye et al., 2019; Wang et al., 2021). Many resistant accessions of Chinese wheat landraces continually display APR to stripe rust in the field, providing a novel resistance resource for the breeding of wheat cultivars with durable resistance to stripe rust. Therefore, it is necessary and important to identify and develop new durable high-level APR resistance genes against stripe rust.

Gaoxianguangtoumai (GX) is a spring wheat landrace from Sichuan Province in southwest China, which is a regional center for oversummering and overwintering of the stripe rust pathogen. This landrace has exhibited a high degree of APR to stripe rust in the field for more than a decade, but little information is available on the genetic basis of resistance in this landrace. The objectives of the present study were to (1) identify the QTL conferring APR to stripe rust in a recombinant inbred line (RIL) population developed from the cross between GX and a susceptible cultivar, “Taichung 29” (TC 29, 2) validate and mendelize the novel QTL in a heterogeneous inbred family (HIF) population, and (3) develop tightly linked Kompetitive allele-specific PCR (KASP) markers for use in marker-assisted selection in breeding programs.

## Materials and Methods

### Plant Materials and Races

The Chinese wheat landrace GX (accession number ZM7854 in National Germplasm Bank, China (NGBC) and AS1579 in Triticeae Research Institute, Sichuan Agricultural University) originating from Gao County, a county of Sichuan Province (28°26′N, 104°31′E). Because of high level of resistance to stripe rust for more than a decade, GX was crossed (as the female parent) with the highly stripe rust susceptible wheat cultivar TC 29. In total, 249 F_6:8_ (F_6_, F_7_, and F_8_) RILs derived from an individual F_1_ plant were developed by single-seed descent. A KASP marker, *KP2A_36.85* which was located around the peak of *QYr.GX-2AS*, was used to identify heterozygous lines from an F_8_ generation of RIL. Through a single heterozygous plant was selected and selfed (Tuinstra et al., 1997), a HIF population of 130 individuals was generated for validating the *QYr.GX-2AS*. The scheme for developing the genetic populations was showed in Supplementary Figure S1. A collection of 325 Chinese wheat landraces was genotyped with the 55K single-nucleotide polymorphism (SNP) array and further was used for marker haplotype analysis (Zhou et al., 2017). A panel of 109 Sichuan wheat cultivars was used to determine the polymorphism of markers tightly linked with *QYr.GX-2AS*. The highly stripe rust susceptible wheat cultivars “Mingxian 169,” “SY95-71,” and “Avocet S” (AvS) were used as susceptible controls in seedling and adult-plant tests throughout the study. Here, SY95-71 is a spring wheat line, selected from hexaploid triticale/wheat followed by backcrossing with wheat (Eronga 83/Fan6∥Fan6; Shu et al., 1999). The line has been widely used in China as a highly susceptible stripe rust spreader genotype or susceptible control. The *Pst* races (comprising CYR32, CYR33, CYR34, G22-14, Su11-4, Su11-5, and Su11-7; Wu et al., 2016b; Huang et al., 2018) were kindly provided by the Plant Protection Institute of the Gansu Academy of Agricultural Sciences, Gansu, China.

### Evaluation of Resistance to Stripe Rust

Seedling tests to evaluate the stripe rust resistance of GX and TC 29 were conducted in a greenhouse using two prevalent Chinese *Pst* races (CYR32 and CYR34). Five plants of each line were sown in a plastic pot filled with nutrient soil and grown in a controlled environment in the greenhouse. Seedlings were inoculated at the two-leaf stage with each *Pst* race in accordance with the protocol of Hickey et al. (2012). Inoculated plants were placed in a dew chamber at 10°C and 100% relative humidity for 24h in the dark, and then moved to separate growth chambers at 15–16°C with 12–14h of light daily. When the susceptible control “Mingxian 169” showed full sporulation, the infection type (IT) on the second leaf (approximately 15–18days after inoculation) was scored using a 0–9 scale (Line and Qayoum, 1992). Plants with IT scores of 1 to 6 were considered resistant, whereas plants with IT scores of 7–9 were considered susceptible.

Assessments of adult-plant stripe rust responses were conducted at the Chongzhou Experimental Station (30°33′N, 103°39′E), Sichuan Agricultural University, Chengdu, China. The F_6:8_ RILs population and the parental lines were evaluated for APR to stripe rust during the 2017–2018, 2018–2019, and 2019–2020 growing seasons (referred to as CZ2018, CZ2019, and CZ2020, respectively). The HIF population of 130 individuals was evaluated for APR to stripe rust during the 2020–2021. The phenotype data of HIF population were used for Chi-Squared analysis (3:1 ratio) and genetic mapping. In all tests, 20 seeds of each line were planted in rows 2m in length and spaced 30cm apart, with individual plants spaced 10cm apart. The susceptible cultivar TC 29 was planted in every 20th row as a susceptible control. To provide inoculum for infection, the susceptible cultivars SY95-71 and AvS were planted around the perimeter of the experimental area as spreaders. Artificial inoculation was conducted using a mixture of currently predominant *Pst* races in China (comprising CYR32, CYR33, CYR34, G22-14, Su11-4, Su11-5, and Su11-7). Stripe rust response was first recorded by scoring the IT and disease severity (DS) when the susceptible checks SY95-71 and AvS showed more than 80% DS and was followed by two additional evaluations at 7day intervals (i.e., three evaluations in total) for three randomly selected individual plants. The IT was recorded based on the 0–9 scale of Line and Qayoum (1992). The DS was scored as the percentage infected leaf area (0, 5, 10, 20, 40, 60, 80%, or 100%) in accordance with the Chinese National Standard, GB/T 15797-2011. The final DS (FDS) was used for phenotypic analysis.

### Genotyping, Linkage Map Construction, and QTL Analysis

Genomic DNA was extracted from a single plant for each line of the wheat materials using the cetyltrimethylammonium bromide method (Stewart and Via, 1993). The two parents (GX and TC 29) and the 117 RILs were genotyped using the Axiom^®^ Wheat 55K SNP array (53,036 markers) by the China Golden Marker Biotechnology Company Ltd. (Beijing, China). Monomorphic and SNP loci with a minor allele frequency less than 0.3 were excluded with further analysis (Ma et al., 2019). Polymorphic SNP markers were used to remove redundant markers in the binning step using the BIN function, with the parameters missing rate=20% and distortion value=0.01, implemented in QTL IciMapping v4.2 (Wang et al., 2019b). The binned markers were used for linkage map construction using the Kosambi mapping function (Kosambi, 1944) with JoinMap v4.0 (Van Ooijen, 2006). Mapping of QTL was performed using QTL IciMapping v4.2 based on inclusive composite interval mapping with the preset parameters Step=1cM, *value of p* for entering variables (PIN)=0.001, and logarithm of the odds (LOD)=2.5.

To determine the effects of the QTL, the RILs were divided into four groups based on the presence/absence of the most closely linked flanking markers of *QYr.GX-2AS* and *QYr.GX-7DS*. In addition, the epistatic interactions between *QYr.GX-2AS* and *QYr.GX-7DS* were identified in RILs using QTL IciMapping v4.2 based on inclusive composite interval mapping of digenic epistatic QTL (ICIM-EPI) functionality with the preset parameters Step=1cM, value of p for entering variables (PIN)=0.0001, and LOD=5.

### Haplotype Analysis

Haplotype analysis was performed to identify haplotype variants for *QYr.GX-2AS* in a collection of 325 Chinese wheat landrace accessions (Zhou et al., 2017; Ye et al., 2019). The informative markers linked to *QYr.GX-2AS* were screened using the Wheat 55K or Wheat 660K SNP arrays in accordance with the method described by Long et al. (2021). The SNP genotype data and the phenotype data (FDS) were obtained from recently published studies (Cheng et al., 2019; Long et al., 2019; Yao et al., 2019, 2020; Ye et al., 2019; Wang et al., 2021). Haplotype variants were detected using Haploview v4.2.^1^ The haplotypes detected in at least 10 accessions were considered to be major haplotypes. Boxplots were generated to display the average FDS of accessions carrying the different haplotypes. Haplotype data were combined with provenance information to examine the geographic distribution of the superior haplotypes in the 10 major agro-ecological production zones of Chinese wheat landraces.

### Exome Capture Sequencing, Development of KASP Markers, and Genetic Mapping

Genomic DNA of the resistant parent GX was sequenced using the wheat exome capture sequencing protocol described by Dong et al. (2020). The raw sequence data have been submitted to GenBank under Bioproject no. PRJNA734801. The sequence variants were identified using the variant calling pipeline GATK4 (Heldenbrand et al., 2019). After QTL mapping, random SNPs in the target region to *QYr.GX-2AS* from the Wheat 55K array and exome capture sequencing were selected and converted to KASP markers using the PolyMarker online tool (Ramirez-Gonzalez et al., 2015). The specific KASP markers were used to screen the parents and a paired of NIL (selected from HIF population with a common genetic background but differing in *QYr.GX-2AS*) to confirm polymorphism before genotyping in the HIF population. The KASP assays were performed in 96-well format as 10μl reactions containing 2μl of 50–100ng genomic DNA, 5μl of HiGeno 2× Probe Mix B, 0.24μM of each forward primer, 0.6μM of the common reverse primer, and double distilled water to make up the volume to 10μl. Each PCR was conducted using the BIO-RAD CFX96 qPCR system. Thermocycling was performed with a touchdown protocol: 95°C for 10min; 95°C for 20s and 61°C (−0.6°C per cycle) for 40s for 10cycles; and 95°C for 20s and 55°C for 40s for 38cycles. Data analysis was performed manually using BIO-RAD CFX96 Manager 3.1.

The polymorphic KASP markers were used for validating the *QYr.GX-2AS* in the HIF population of 130 individuals. Linkage analysis was performed using JoinMap v4.0 (Kyazma BV, Wageningen, Netherlands; Van Ooijen, 2006) with a LOD threshold of 3.0. The Kosambi map function (Kosambi, 1944) was used to convert the recombination fractions to centi-Morgans. The linkage map was drawn using Mapdraw v2.1 (Liu and Meng, 2003). Three tightly linked markers for *QYr.GX-2AS* were further assessed in 109 wheat cultivars grown in Sichuan for checking the usefulness of the newly developed KASP markers for marker-assisted selected.

### Data Analyses

Best linear unbiased prediction (BLUP) values for each RIL, ANOVA, Pearson’s correlation coefficients, and broad-sense heritability (*H*^2^) estimates were calculated using the “AOV” tool implemented in QTL IciMapping v4.2^2^ (Wang et al., 2019b). The Chi-squared (*χ*^2^) test with Excel 2016 was used to evaluate the goodness-of-fit for phenotype data of RILs population (1:1 ratio and 3:1 ratio) and HIF population (3:1 ratio). Student’s *t*-tests (*p*<0.05 and 0.01) were conducted with SPSS Statistics v17.0 (IBM Corp., Armonk, NY, United States) to evaluate the significance of differences between the two groups.

## Results

### Stripe Rust Response of the Parents and RILs

**Figure 1:**
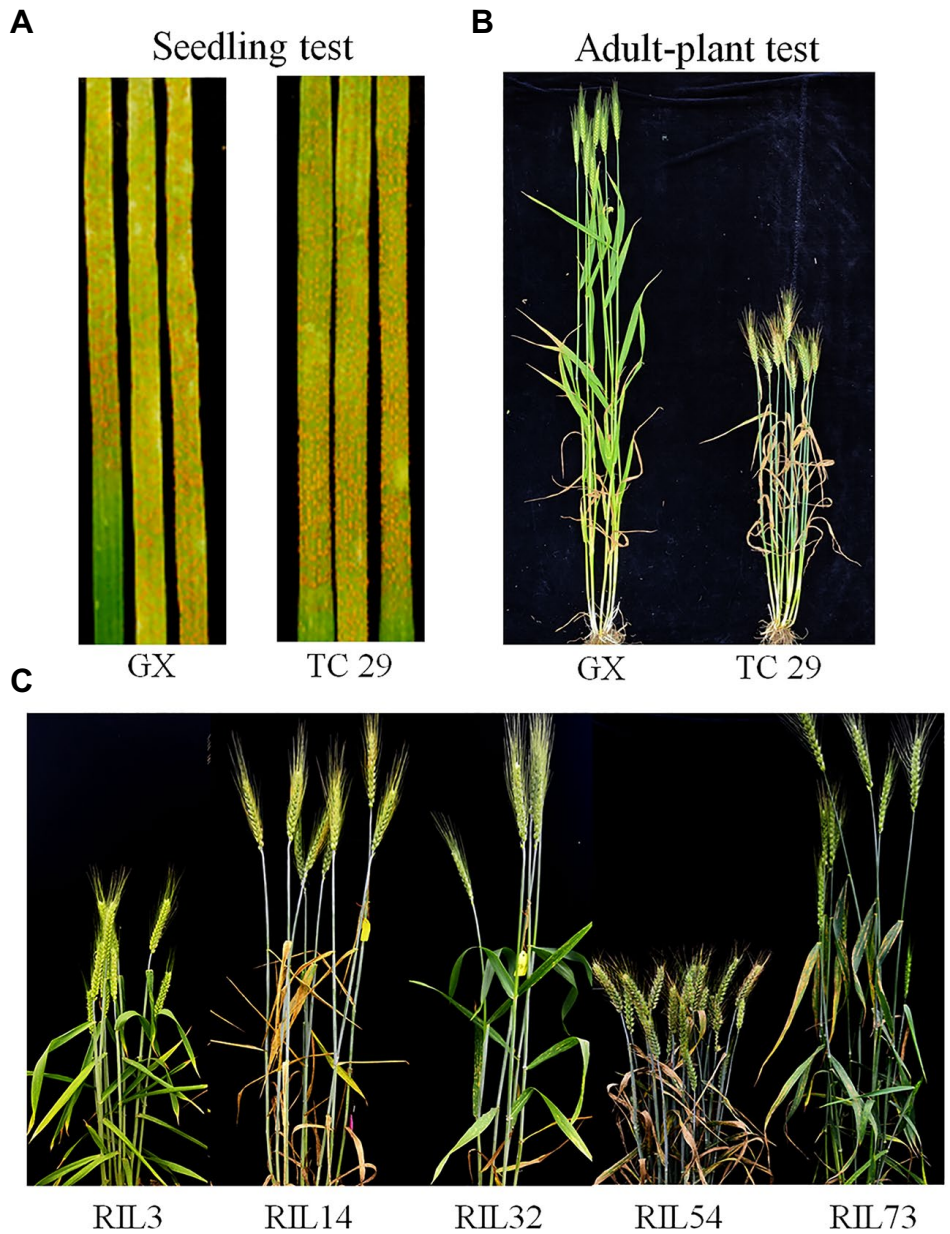
Stripe rust response of the resistant parent Gaoxianguangtoumai (GX) and susceptible parent Taichung 29 (TC 29) with CYR34 at the seedling stage **(A)** and mixture *Pst* at the adult-plant stage **(B)**; Stripe rust response of the randomly selected recombinant inbred lines (RILs) of lines in the field **(C)**.

**Figure 2:**
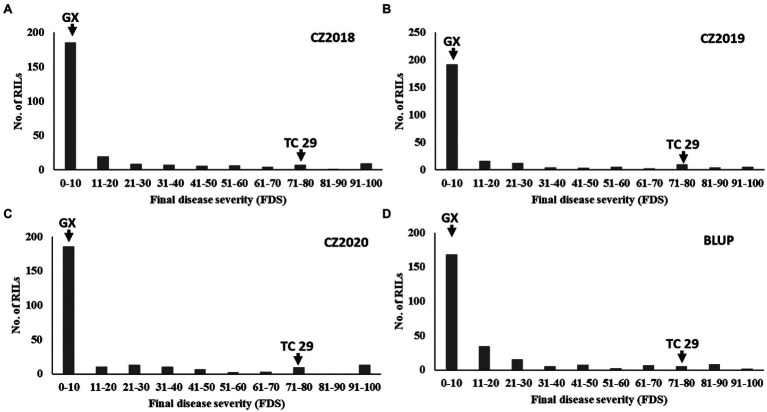
Frequency distributions of the final disease severity (FDS) for the recombinant inbred lines (RILs) population derived from Gaoxianguangtoumai (GX)×Taichung 29 (TC 29) at Chongzhou in 2018 **(A)**, 2019 **(B)**, 2020 **(C)**, and best linear unbiased prediction (BLUP) values **(D)**.

Plants of GX were susceptible (IT=8) to CYR32 and CYR34 at the seedling stage (Figure 1A) but exhibited strong resistance (IT=3, FDS<10%) to mixed *Pst* races (comprising CYR32, CYR33, CYR34, G22-14, Su11-4, Su11-5, and Su11-7) at the adult-plant stage in three crop seasons from 2018 to 2020 (Figures 1B, 2; Supplementary Table S1), indicating that GX has effective APR to these prevalent Chinese *Pst* races. In all three environments, the average FDS of RILs for GX×TC 29 was 12.5–15.7% in the field tests, and the distributions were skewed toward resistance (Figure 2). A total of 200 homozygous resistant lines (IT ≤6) were consistently observed in the 249 RILs in all three field trials, and 142 lines of them showed high resistance similar to GX (IT ≤3). In addition, 25 homozygous susceptible lines (IT ≥7) were consistently observed in all three field trials. According to the homozygous phenotypes, the distribution of F_6:8_ families was not fit the expected ratios for a single gene (1:1 ratio; *χ*^2^=136.11, *p*<0.001) and two genes (3:1 ratio; *χ*^2^=23.15, *p*<0.001). The result indicated that the high level of resistance in GX was controlled by multiple genes (Figures 1C, 2; Supplementary Table S1). Broad-sense heritability (*H*^2^) was 96.7% for FDS in all tests (Table 1). Correlation coefficients (*R*^2^) for FDS of the RILs among the different environments were significant (*p*<0.01) and ranged from 0.82 to 0.95 (Supplementary Table S2).

**Table 1:** The summary of final disease severity (FDS) data for the recombinant inbred lines (RILs) population from the Gaoxianguangtoumai (GX)×Taichung 29 (TC 29) recorded in the fields at Chongzhou in 2018–2020.

Environments	Parents		RILs population				
	GX	TC 29	Min-max	Mean	SD	CV	*H*^2^ (%)
CZ2018 (%)	5.0	80.0	0–100	14.0	25.2	1.8	
CZ2019 (%)	6.7	80.0	0–100	12.5	23.7	1.9	
CZ2020 (%)	5.0	80.0	0–100	15.7	27.0	1.7	
BLUP (%)	7.1	73.2	2.1–91.0	14.6	21.2	1.5	96.7

*SD, standard deviation; CV, coefficient of variation; and H^2^, broad-sense heritability*.

### Linkage Map Construction and QTL Analysis

A total of 1,871 markers were used to construct the linkage map which spanning a total length 2,799.12cM for the GX×TC 29 population (Supplementary Table S3). The A, B, and D genomes included 681 (36.40%), 669 (35.76%), and 521 (27.85%) markers covering lengths of 911.04, 855.71, and 1,032.37cM with average marker intervals of 1.34, 1.28, and 1.98cM, respectively (Supplementary Table S3).

**Figure 3:**
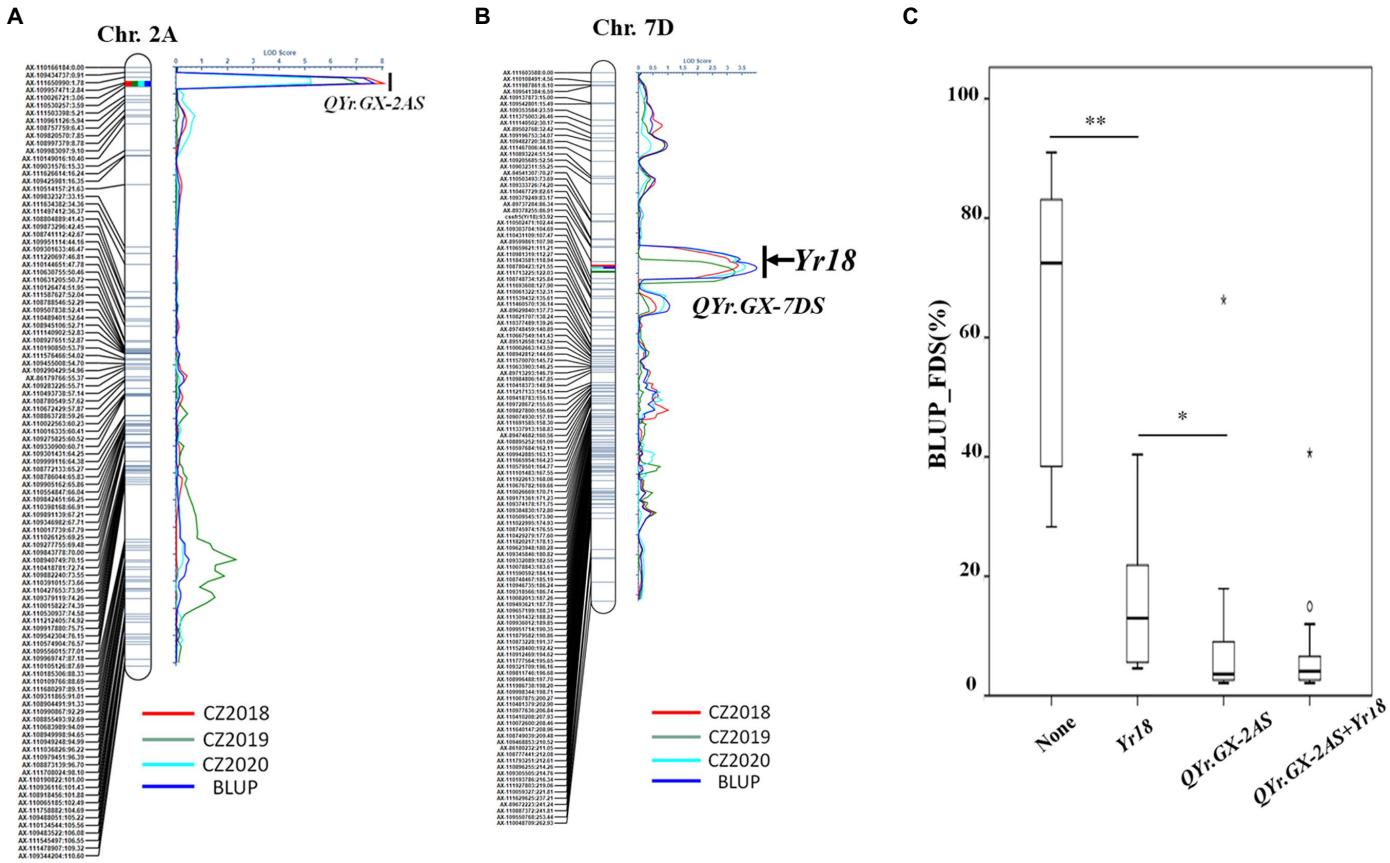
QTL conferring adult plant stripe rust resistance detected by inclusive composite interval mapping (ICIM) in the recombinant inbred lines (RILs) population from Gaoxianguangtoumai (GX)×Taichung 29 (TC 29). Graphical displays of QTL **(A)** *QYr.GX-2AS* and **(B)** *QYr.GX-7DS* detected on chromosome 2A and 7D based on the final disease severity (FDS) from three field trials and best linear unbiased prediction (BLUP) data. The box plots for final disease severity (FDS) based on the best linear unbiased prediction (BLUP) data associated with the two loci (*QYr.GX-2AS* and *Yr18)* and their combination in the Gaoxianguangtoumai (GX)×Taichung 29 (TC 29) recombinant inbred lines (RILs) population **(C)**. *indicate significant at *P*=0.05; **indicate significant at *P*=0.01.

Two high quality QTL, conferring APR to *Pst* races, was screened through further analysis (Table 2; Figures 3A,B). The most significant QTL, designated *QYr.GX-2AS*, was mapped to the short arm of chromosome 2AS and explained 15.5–27.0% phenotypic variation (Table 2; Figure 3A). The other QTL, designated *QYr.GX-7DS* and explaining 11.5–13.5% phenotypic variation, was located on the short arm of chromosome 7D where this gene overlaps with *Yr18* (Krattinger et al., 2009). The genetic distances analysis showed SNP markers *cssfr5* and *AX-110502471* flanking *QYr.GX-7DS* were 3.1cM and 5.4cM, respectively (Table 2; Figure 3B). Results indicated that it was highly likely that *QYr.GX-7DS* corresponded to *Yr18*.

**Table 2:** Quantitative trait loci (QTL) for stripe rust resistance detected in the recombinant inbred lines (RILs) population from the Gaoxianguangtoumai (GX)×Taichung 29 (TC 29) using final disease severity (FDS) data across three environments and best linear unbiased prediction (BLUP) values.

QTL	Environment	Trait	Chromosome	Left Marker	Right Marker	Chromosome interval (cM)	LOD	PVE (%)	Resistance source
*QYr.GX-2AS*	CZ2018	FDS	2AS	*AX-109957471*	*AX-110026721*	2.8–3.1	8.1	27.0	GX
	CZ2019			*AX-109957471*	*AX-110026721*	2.8–3.1	7.1	17.1	
	CZ2020			*AX-109957471*	*AX-110026721*	2.8–3.1	5.2	15.5	
	BLUP			*AX-109957471*	*AX-110026721*	2.8–3.1	7.7	21.8	
*QYr.GX-7DS*	CZ2018	FDS	7DS	*cssfr5* (*Yr18*)	*AX-110502471*	93.9–102.4	3.4	11.6	GX
	CZ2019			*cssfr5* (*Yr18*)	*AX-110502471*	93.9–102.4	3.2	11.5	
	CZ2020			*cssfr5* (*Yr18*)	*AX-110502471*	93.9–102.4	3.6	12.4	
	BLUP			*cssfr5* (*Yr18*)	*AX-110502471*	93.9–102.4	4.0	13.5	

Clearly, the RILs that carried one of the QTL showed a lower FDS than those without any QTL (average FDS=63.4%; Figure 3C). The RILs carrying only *QYr.GX-7DS* showed 14.8% of the average FDS, whereas average FDS of lines with only *QYr.GX-2AS* was 9.3%. The lines with two QTL had the highest resistance level (average FDS=7.06%; Figure 3C), similar to that of GX. In addition, the epistatic interaction between *QYr.GX-2AS* and *QYr.GX-7DS* could be significantly detected in two field trials and Busing the ICIM-EPI functionality of the QTL IciMapping v4.2 (Supplementary Table S4). These results indicated that the high-level resistance in GX was contributed by these two QTL through additive and epistatic interactions, where *QYr.GX-2AS* provided relatively stronger resistance to *Pst* races than *QYr.GX-7DS*.

### Haplotype Analysis of *QYr.GX-2AS*

**Figure 4:**
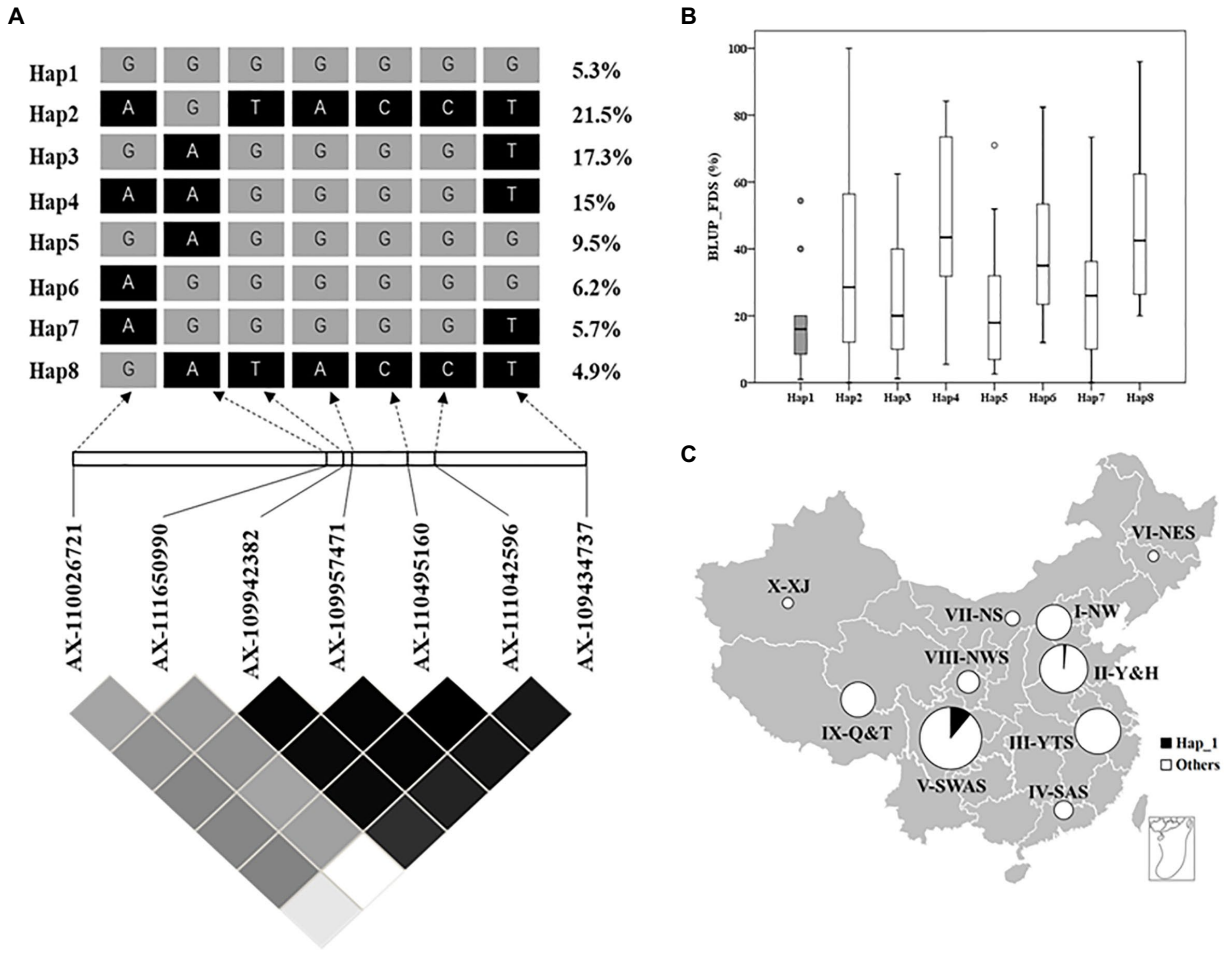
Haplotype analysis of *QYr.GX-2AS* associated with stripe rust resistance in 325 Chinese wheat landraces. **(A)** LD heat map surrounding *QYr.GX-2AS*. The number on the right shows the distribution frequency of eight haplotypes in these Chinese wheat landraces. **(B)** Boxplot displays the mean final disease severity of the accessions carrying different haplotypes. **(C)** Frequencies of resistance allele of *QYr.GX-2AS* in Chinese wheat landraces in 10 major agro-ecological production zones of China.

To assess the distribution of *QYr.GX-2AS* among 325 Chinese wheat landraces, the favorable haplotype was identified by haplotype analysis and seven SNP markers tightly linked to *QYr.GX-2AS* were screened from the Wheat 55K or 660K SNP arrays (Figures 4A–C). Eight major haplotypes (*n*>10) were detected in the panel (Figures 4A,B). GX and 15 other accessions clustered with Hap1 (Supplementary Table S5), which showed a frequency of about 5.3% in the total population (Figure 4A). Almost all accessions carrying Hap1, except one from Henan, were collected from Sichuan. The accessions carrying Hap1 showed 18.4% of the average FDS and thus were more strongly resistant to stripe rust than those accessions carrying other haplotypes (Hap2=37.2%, Hap3=24.1%, Hap4=47.7%, Hap5=21.5%, Hap6=39.0%, Hap7=27.0%, and Hap8=47.6%; Figure 4C). The above results suggested that Hap 1 was the favorable haplotype of *QYr.GX-2AS* and relatively rare in Chinese wheat landraces.

### Validation and Mapping of *QYr.GX-2AS*

**Figure 5:**
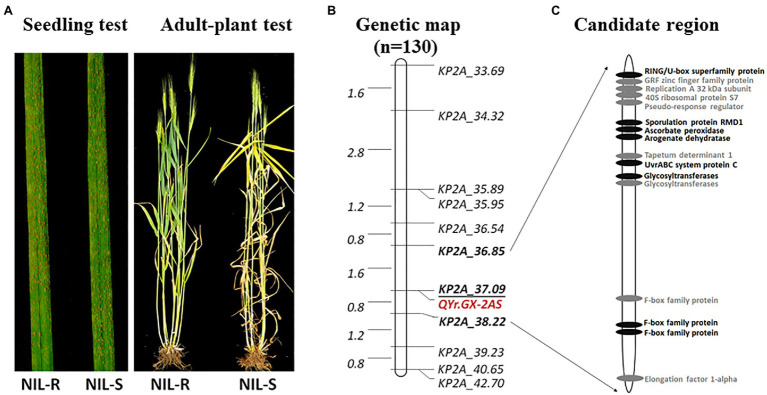
Stripe rust response of the near-isogenic lines with mixture *Pst* at the adult-plant stage in the field **(A)**, genetic map of chromosomes 2AS showing locations of stripe rust resistance genes *QYr.GX-2AS* based on the heterogeneous inbred family (HIF) population **(B)**, predicted genes in IWGSC RefSeq v1.1, highlight in black showed non-synonymous variants between Gaoxianguangtoumai (GX) and Chinese Spring in the exon regions **(C)**.

*QYr.GX-2AS* was further mapped finely using newly KASP markers developed from SNPs screened by exome capture sequencing and the Wheat 55K array. Eleven markers were confirmed to be polymorphic between GX and TC 29 (Supplementary Table S6). Combined with the KASP marker *KP2A_36.85* for *QYr.GX-2AS* and the marker *cssfr5* for *Yr18*, the HIF population of 130 individuals with a single locus *QYr.GX-2AS* was developed from a heterozygous plant (IT=4) in the F_8_ generation of RILs (Supplementary Figure S1). No significant phenotypic differences were observed in the HIF population, except for APR to stripe rust (Figure 5A). With regard to stripe rust response in the field test, the HIF population could be clearly classifiable into 97 resistant (IT=3–4) and 33 susceptible (IT=8–9) individuals, which fits the expected ratio (3:1) for a single Mendelian factor (chi-square goodness-of-fit test, *χ*^2^=0.01, *p*=0.92; Supplementary Table S7). Using the newly developed 11 KASP markers (Supplementary Table S7) to construct the genetic map, *QYr.GX-2AS* was screened in 1.37Mb interval between the KASP marker *KP2A_36.85* and *KP2A_38.22* and co-segregated with the *KP2A_37.09* (Figure 5B).

### Validation of KASP Markers for Marker-Assisted Selection

The molecular identification of 109 Chinese wheat cultivars was tested with three KASP markers *KP2A_36.85* (G/A), *KP2A_37.09* (A/C), and *KP2A_38.22* (G/A; Supplementary Figure S2; Supplementary Table S8), which suggested that most of the cultivars could be amplified susceptible-specific alleles and showed 85.3, 99.1, and 95.4% polymorphism, respectively (Supplementary Table S8). Based on the above results, three KASP markers were valuable to apply *QYr.GX-2AS* in wheat breeding by marker-assisted selection.

## Discussion

It is the highest priorities to develop durable resistance to *Pst* races in wheat breeding during the past decade (Chen, 2013). A large number of genes or QTL that confer various degrees of APR to stripe rust have been identified (Chen, 2013), but most only have minor effects on stripe rust response and are therefore difficult to use in breeding. Thus, the identification of new high quality *Yr* genes or QTL with APR is useful in wheat breeding. The Chinese wheat landrace GX has displayed a high degree of APR to stripe rust in the field for more than a decade in southwest China. Two QTLs conferring APR to *Pst* races tested were identified in GX, tentatively named as *QYr.GX-2AS* and *QYr.GX-7DS*, and mapped on chromosome2AS and 7DS, respectively. In addition, the *QYr.GX-2AS* had a large effect in the reduction of stripe rust severity at adult-plant stages, which would be expected to have a great potential to pyramid this QTL with other *Yr* gene/QTL to develop wheat cultivars with high-level and durable resistance to *Pst* races.

QTL analysis is a useful procedure to reveal possible multiple loci when analyzing complex genetic traits, such as APR to stripe rust, in resistant germplasm. However, this procedure only allows approximate mapping of the QTL (Tanksley and Hewitt, 1988) owing to the heterogeneity in genetic backgrounds. The confidence interval of many QTL spans a considerable genetic distance and, as a result, molecular markers for these QTL may not be reliably used in marker-assisted selection. As a strategy for accurate mapping of QTL in genetic analysis, HIF populations that allow the conversion of a quantitative trait into a Mendelian factor have been widely used for fine mapping and cloning of many important QTL in wheat, such as *Yr18* (Krattinger et al., 2009), *Yr36* (Fu et al., 2009), *Fhb1* (Su et al., 2019), and *Fhb7* (Wang et al., 2020). In the present research, a HIF population targeting *QYr.GX-2AS* was developed based on the method of heterogeneous inbred family analysis (Tuinstra et al., 1997). Members of this population were unambiguously classified as either resistant or susceptible and fitted the expected ratio (3:1) for a single Mendelian factor; thus, accurate mapping of the locus was possible. Analysis of the HIF population revealed that *QYr.GX-2AS*, flanked by *KP2A_36.85* and *KP2A_38.22*, was located in the interval 36.85Mb to 38.22Mb on chromosome 2AS. One KASP marker co-segregating with the targeted locus was successfully developed for marker-assisted selection.

**Figure 6:**
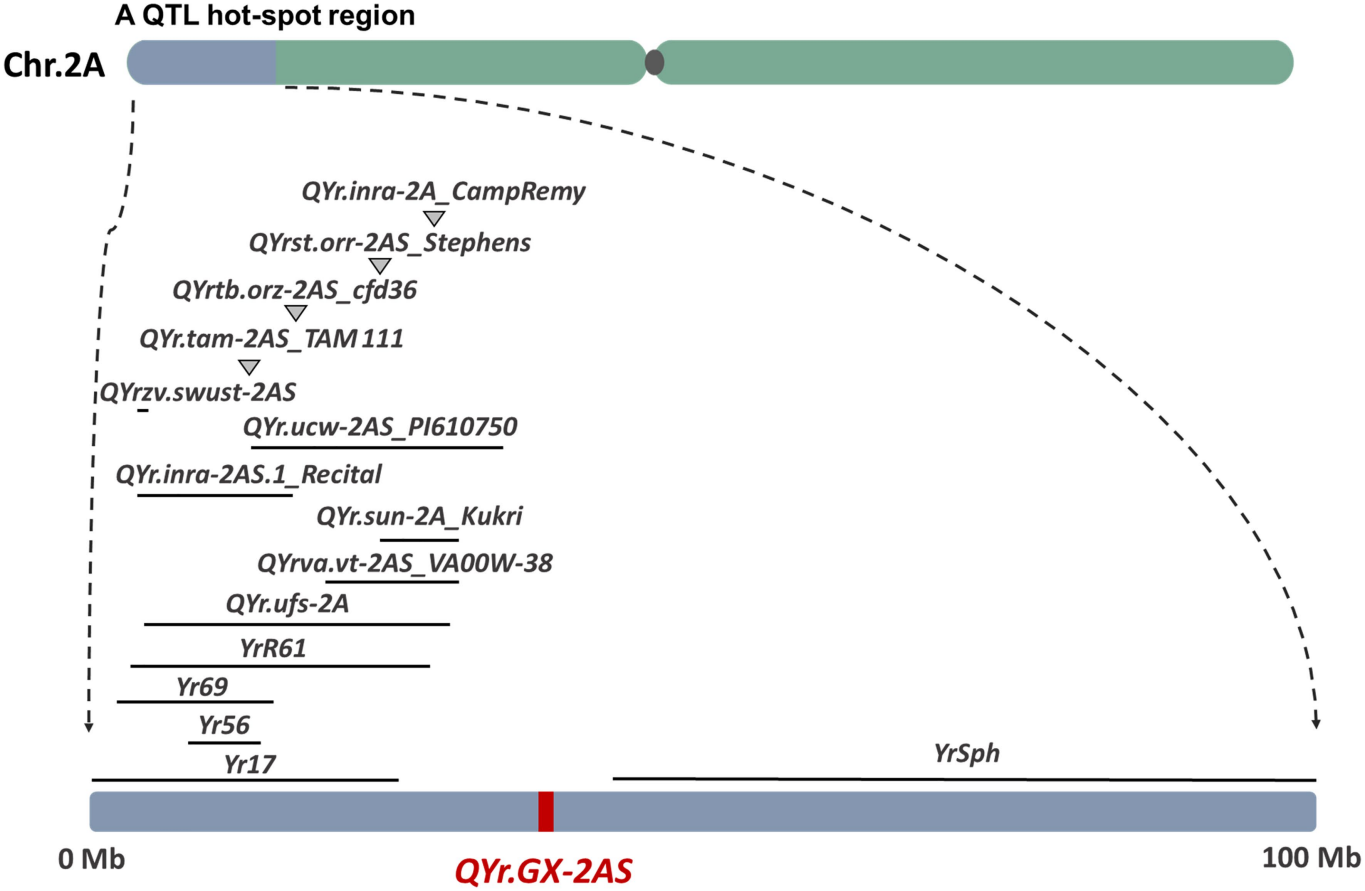
Comparison of *QYr.GX-2AS* with previously identified genes/QTL (from biparental population) for resistance to stripe rust based on the reference genome of bread wheat (IWGSC, RefSeq v1.0).

Several genes that confer resistance to stripe rust have been identified on wheat chromosome 2AS, including *Yr17* (Bariana and Mcintosh, 1993), *Yr56* (Bansal and Bariana 2014), *Yr69* (Hou et al., 2016), *YrR61* (Hao et al., 2011), and *YrSph* (Chen et al., 2012; Figure 6 and Supplementary Table S9). The genes *Yr17*, *Yr69*, and *YrSph* confer ASR to stripe rust. Although recent studies suggest that *Yr17* also confers APR to stripe rust in the field, *QYr.GX-2AS* is likely to differ from *Yr17* because accessions of the Chinese wheat landrace GX that lack the 2N alien segment carry *Yr17*. *Yr56* is a major gene conferring APR to stripe rust that was identified in the Australian durum wheat cultivar “Wollaroi.” *Yr56* is flanked by *Xsun167* (*wPt-4,197*) and *Xsun168* (*wPt-9104*; Bansal and Bariana 2014), which corresponds to the “Chinese Spring” physical map region between 8.35Mb and 14.28Mb. *YrR61*, corresponding to the major-effect QTL *QYr.uga-2AS_26R61* conferring APR to stripe rust, was identified from the soft red winter wheat cultivar “Pioneer” and is flanked by the markers *Xbarc124* (3.78Mb) and *Xgwm359* (28.20Mb; Hao et al., 2011). Clearly, both *Yr56* and *YrR61* are located distant from *QYr.GX-2AS*. In addition, at least 20 QTL have been reported on chromosome 2AS, and most of them are located at a QTL hot-spot region in the distal end of 2AS (<30Mb; Figure 6). For example, the QTL *QYr.tam-2AS_TAM 111* (Basnet et al., 2014) confer ASR to stripe rust. *QYr.ufs-2A* (Agenbag et al., 2012), *QYrst.orr-2AS_Stephens* (Vazquez et al., 2012), and *QYr.sun-2A_Kukri* (Bariana et al., 2010) were all flanked by the basis of a common DArT marker *XwPt-0003*, which were nearly with the *QYrva.vt-2AS_VA00W-38* (Christopher et al., 2013) corresponds to the “Chinese Spring” physical map region 29.94Mb. *QYrtb.orz-2AS* (Vazquez et al., 2015) and *QYr.inra_2AS.1_Recital* (Dedryver et al., 2009) were located in 2AS close to marker *Xcfd36* (about 16.63Mb) which are homeologous to the *Yr17* introgression. The *QYr.ucw-2AS_PI610750* (Lowe et al., 2011), contributed by the synthetic derivative PI610750, is flanked by the *XwPt-3896* (13.14Mb) and *Xwmc177* (33.70Mb). *QYr.inra-2A_CampRemy* from Camp Remy (Mallard et al., 2005) is located by the *Xgwm382a* and *Xgwm359* (about 28.20Mb). *QYrzv.swust-2AS* (Zhou et al., 2021) flanked by *IWB7877* and *IWB72720* is derived from the wild emmer wheat (*T. dicoccoides*) accession Zavitan, corresponding to the “Chinese Spring” physical map region between 5.25Mb and 5.33Mb. Similarly, the other QTL identified by GWAS is located in different regions from *QYr.GX-2AS* on chromosome 2AS, expect for a minor locus *QYr.wsu-2A.1_IWA2526* (about 36.63Mb). Hence, the large-effect QTL *QYr.GX-2AS* identified in the present study is unlikely to be the previously reported QTL. Anyway, the most powerful evidence still is gene sequencing on the target region after cloning *QYr.GX-2AS*.

According to gene annotation information in IWGSC RefSeq v1.1, 16 predicted genes are located in the candidate region for *QYr.GX-2AS* (Figure 5C; Supplementary Table S10). None of these genes is a classic NBS-LRR resistance gene. In addition, no annotations accorded with the protein types encoded by the APR genes *Yr18* (ABC transporter), *Yr36* (kinase-START), and *Yr48* (hexose transporter), implying that the candidate gene for *QYr.GX-2AS* might differ from known stripe rust resistance genes. Combined with exon sequencing data, eight predicted genes showed non-synonymous variants between GX and “Chinese Spring” in exon regions, including a RING/U-box, ascorbate peroxidase, glycosyltransferases, and F-box family protein, that may be involved in disease resistance. For confirmation of the candidate gene and cloning of *QYr.GX-2AS*, fine mapping to narrow the candidate interval will be performed using a large HIF population in future work.

## Data Availability Statement

The datasets presented in this study can be found in online repositories. The names of the repository/repositories and accession number(s) can be found in the article/Supplementary Material.

## Author Contributions

YW and FL are responsible for the experiment, analyzed the data, and drafted the manuscript. FG, FY, LL, XZ, LD, YW, and HL carried out the phenotypic evaluation. WL, QJ, YW, JM, PQ, MD, and YZ provided the resources and technique guidance. HK, YJ, and GC designed and carried out the experiment, formulated the questions, analyzed the data, and revised the manuscript. All authors have reviewed and approved the final manuscript.

## Funding

This research was supported by the National Key Research and Development Program of China (2016YFD0100100), the International Science and Technology Cooperation and Exchanges Programs of Science and Technology Department of Sichuan Province (2019YFH0063), and the Applied Basic Research Programs of Sichuan Province (2021YJ0297).

## Conflict of Interest

The authors declare that the research was conducted in the absence of any commercial or financial relationships that could be construed as a potential conflict of interest.

## Publisher’s Note

All claims expressed in this article are solely those of the authors and do not necessarily represent those of their affiliated organizations, or those of the publisher, the editors and the reviewers. Any product that may be evaluated in this article, or claim that may be made by its manufacturer, is not guaranteed or endorsed by the publisher.

## Footnotes

^1^ http://www.broad.mit.edu/mpg/haploview/

^2^ http://www.isbreeding.net
